# Diagnostic Performance and Clinical Utility of Automated Plasma Amyloid-β 1-42/1-40 Assay

**DOI:** 10.3390/diagnostics16121767

**Published:** 2026-06-08

**Authors:** Seseung Kim, Seok Ryun Kwon, Joon Hee Lee, Kyunghoon Lee, Sang Hoon Song, Junghan Song

**Affiliations:** 1 Department of Laboratory Medicine, Seoul National University Bundang Hospital, Seongnam 13620, Republic of Korea; kimseseung.bio@gmail.com (S.K.); songjhcp@gmail.com (J.S.); 2 Department of Laboratory Medicine, Seoul National University College of Medicine, Seoul 03080, Republic of Korea; kwonseokryun@gmail.com (S.R.K.); cloak21@snu.ac.kr (S.H.S.); 3 Department of Laboratory Medicine, Seoul National University Hospital, Seoul 03080, Republic of Korea

**Keywords:** Alzheimer’s disease, mild cognitive impairment, amyloid beta, biomarker, Aβ42/40 ratio, immunoassay, HISCL

## Abstract

**Background**: Blood-based biomarkers offer an accessible alternative to cerebrospinal fluid or positron emission tomography (PET) imaging for Alzheimer’s disease (AD) screening and diagnosis. This study evaluated the diagnostic performance of the fully automated HISCL plasma Aβ42/40 assay in a real-world clinical setting. **Methods**: We retrospectively enrolled 127 participants, stratified into cognitively normal (CN), mild cognitive impairment (MCI), AD, and Non-AD subgroups. Plasma Aβ42/40 levels were quantified using the HISCL and Simoa platforms. Additionally, plasma oligomerized Aβ (OAβ), glial fibrillary acidic protein (GFAP), and neurofilament light chain (NfL) were measured. **Results**: The HISCL plasma Aβ42/40 ratio was significantly lower in the AD continuum (MCI + AD) compared to the CN subgroup (*p* < 0.001). The HISCL assay demonstrated robust diagnostic performance (AUC = 0.747), yielding a comparably higher AUC value compared to the Simoa Aβ42/40 ratio (AUC = 0.687). Although method comparison showed a proportional difference between HISCL and Simoa, the HISCL assay maintained high discriminative capability. Notably, integrating plasma GFAP and NfL with the HISCL Aβ42/40 ratio significantly enhanced the diagnostic accuracy (AUC = 0.823, *p* = 0.046). Method comparison between heparinized and EDTA plasma in the HISCL assay confirmed assay stability, showing a significant correlation and a regression slope near unity. **Conclusions**: The HISCL plasma Aβ42/40 assay demonstrates reliable diagnostic performance for identifying AD pathology in clinical practice, showing stability across sample types. Furthermore, its combination with neurodegeneration markers significantly improves predictive accuracy, supporting its utility as a robust screening tool and foundational component of future multimarker diagnostic panels.

## 1. Introduction

As of 2024, the total cost of dementia management in South Korea is estimated at approximately 24 trillion KRW (approximately 18 billion USD), with projections indicating that these costs will double every decade due to the rapidly aging population [1]. Evidently, this escalating financial expenditure imposes a substantial burden on both the national healthcare system and individual households.

It is well established that the early diagnosis of Alzheimer’s disease (AD) is crucial for slowing disease progression and mitigating its substantial socioeconomic burden [2]. In particular, with the emergence of the Alzheimer’s disease continuum concept [3], the key role of biomarkers in early detection has gained significant attention. In particular, blood-based biomarkers (BBMs) are less invasive and have lower costs, providing more time-efficient measurements than cerebrospinal fluid (CSF) or molecular neuroimaging biomarkers [4]. Notably, the incorporation of BBMs into the 2024 Alzheimer’s Association diagnostic criteria highlights a broad consensus regarding their clinical utility [5].

Among these, biomarkers related to amyloid-β (Aβ) pathology are of primary interest due to their role as the earliest detectable indicator in the AD cascade. Pathologically, the aggregation of Aβ peptides, particularly the 42-amino acid isoform (Aβ42), leads to their sequestration into insoluble plaques within the brain; this process is reflected in peripheral blood as a reduction in soluble Aβ42 levels or a decreased amyloid-β 1-42/1-40 (Aβ42/40) ratio, serving as a surrogate marker for cerebral amyloid deposition [6].

Historically, challenges existed due to the low concentrations of target analytes in plasma compared to CSF [7,8]. Particularly, plasma Aβ level shows relatively low difference between patients and healthy controls, approximately 10–15% [9]. However, recent technological advancements have revolutionized this landscape. The introduction of enhanced immunoassay techniques, including electrochemiluminescence assay and single-molecule array (Simoa) assay, as well as liquid chromatography–tandem mass spectrometry, has enhanced analytical sensitivity and specificity [10].

In this study, we evaluated the diagnostic performance of the HISCL β-Amyloid 1-42/1-40 Assay Kit (Sysmex Corporation, Kobe, Japan), utilizing a fully automated chemiluminescence enzyme immunoassay (CLEIA) methodology known for its rapid turnaround time and high throughput capabilities [11]. We compared its diagnostic accuracy against the ultra-sensitive single molecule array (Simoa) assay (Quanterix, Billerica, MA, USA), which currently serves as a high-performance benchmark in blood-based biomarker research, and the AlzOn test (PeopleBio, Seongnam, Republic of Korea), a multimer detection system assay approved by the Korean Ministry of Food and Drug Safety for the selective detection of oligomerized amyloid-β (OAβ) in plasma [12].

## 2. Materials and Methods

### 2.1. Study Population

This study retrospectively enrolled patients who underwent oligomerized amyloid beta (OAβ) testing using heparinized plasma at Seoul National University Bundang Hospital between December 2023 and October 2025. To enable comparative analysis, inclusion was restricted to participants who had accessible residual samples in K2-ethylenediaminetetraacetic acid (EDTA) tubes collected on the same day as the heparinized samples used for routine OAβ testing, thereby ensuring paired EDTA and heparinized samples for each participant. Among the eligible candidates, one participant was excluded due to insufficient residual sample volume, and two other participants were excluded due to an unquantifiable result. Consequently, 127 participants were included in the final analysis.

This study was approved by the Institutional Review Board (IRB) of Seoul National University Bundang Hospital (IRB No. E-2502-954-350). The requirement for written informed consent was waived by the IRB because this study utilized residual samples that were scheduled for disposal following routine clinical analysis.

### 2.2. Clinical Assessment and Classification

Medical records were reviewed to obtain demographic data and results from the Mini-Mental State Examination (MMSE), global Clinical Dementia Rating (CDR), *APOE* genotyping, and amyloid positron emission tomography/computed tomography (PET/CT). Participants were stratified into four diagnostic subgroups based on clinical assessments performed by neurologists or psychiatrists. The Cognitively Normal (CN) subgroup included individuals with no subjective or objective cognitive decline, as well as those diagnosed with subjective cognitive impairment who reported subjective decline but demonstrated no objective memory deficits upon evaluation. The Mild Cognitive Impairment (MCI) subgroup consisted of clinically diagnosed patients; their eligibility was reconfirmed according to conventional criteria revised in 2004 by the International Working Group [13]. Patients presenting with non-amnestic mild cognitive impairment (MCI) were classified into the Non-AD subgroup, given substantial evidence suggesting that this subtype is frequently associated with non-amyloid etiologies rather than AD pathology [14]. For the AD subgroup, the diagnosis was confirmed based on either (1) objective evidence of AD pathology consistent with findings on amyloid-PET/CT, or (2) fulfillment of the criteria for possible major neurocognitive disorder due to Alzheimer’s disease as outlined in the Diagnostic and Statistical Manual of Mental Disorders, 5th edition, text revision (DSM-5-TR) [15]. Specifically, the clinical diagnosis required persistent cognitive decline, defined as cognitive performance falling below −1.5 standard deviations (SDs) on comprehensive neuropsychological tests, or corresponding impairment on the MMSE, recorded on at least two separate occasions. Furthermore, qualified experts verified multi-domain cognitive impairment through a comprehensive neuropsychological assessment, and interference with activities of daily living was verified. The Non-AD subgroup included patients showing cognitive impairment but diagnosed with other etiologies, including Parkinson’s disease (PD), Parkinson-plus syndromes, frontotemporal dementia, vascular dementia, normal pressure hydrocephalus, limbic-predominant amnestic neurodegenerative syndrome, or delirium, as well as those with clear secondary organic causes.

### 2.3. Plasma OAβ Measurement

Blood plasma was collected in lithium heparin-anticoagulated tubes via venipuncture. Plasma OAβ levels were quantified at Seoul National University Bundang Hospital (Seongnam, Republic of Korea) using the AlzOn assay (PeopleBio, Seongnam, Republic of Korea). Heparinized plasma was separated and pretreated according to the manufacturer’s protocol, followed by incubation at 37 °C for 48 h. The resulting chemiluminescent signals were analyzed using the Diamond C system (MicroDigital, Seongnam, Republic of Korea). For each analytical batch, quality control was performed using the manufacturer-supplied OAβ control materials, and runs were accepted only when control values were within the manufacturer-defined acceptance ranges.

### 2.4. Plasma Aβ42, Aβ40, GFAP, and NfL Measurement

Residual plasma samples in EDTA-anticoagulated tubes, paired with the heparinized samples analyzed for OAβ, were used for the measurement of plasma Aβ42, Aβ40, GFAP, and NfL. Plasma Aβ42 and Aβ40 levels were quantified by CLEIA using the HISCL β-Amyloid 1-42 and 1-40 Assay Kits on the HISCL-800 Analyzer (Sysmex Corporation, Kobe, Japan).

The analytical performance of these assays has been characterized previously [16]. Briefly, the limits of quantification (LoQ) were 0.16 pg/mL for Aβ42 and 2.46 pg/mL for Aβ40. Within-run (repeatability) coefficients of variation (CVs) were 1.7–2.0% for Aβ42 and 2.0–3.7% for Aβ40, and within-laboratory (intermediate) CVs were 4.2–5.3% for Aβ42 and 2.3–4.6% for Aβ40. The assays demonstrated high analytical specificity, with cross-reactivity below 0.5% across Aβ peptides of differing lengths and a strong correlation with an immunoprecipitation–mass spectrometry reference method (Pearson’s r = 0.82 for Aβ42 and 0.91 for Aβ40). In the present cohort, all plasma Aβ42 and Aβ40 results were above the respective LoQ and fell within the validated analytical measuring range of the assays; no specimen yielded a value outside the measuring range or required dilution or re-assay.

Assay-level quality control was monitored using the dedicated two-level HISCL β-Amyloid Control (Low and High; lot QNDV-007, Sysmex Corporation), prepared from synthetic Aβ peptides. For every analytical run, both control levels were measured prior to patient samples and verified against the lot-specific target ranges assigned by the manufacturer (Low: Aβ40 86.00–114.00 pg/mL, Aβ42 12.81–16.99 pg/mL; High: Aβ40 160.82–213.18 pg/mL, Aβ42 25.20–33.40 pg/mL). A run was accepted only when both control levels were within their assigned ranges; otherwise, the run was repeated before patient results were released.

Additionally, the Simoa Neurology 4-Plex E multiplex digital immunoassay was performed on the Quanterix HD-X Analyzer (Quanterix, Billerica, MA, USA) to measure plasma GFAP and NfL; quality control for these analytes was performed using the manufacturer-supplied controls according to the manufacturer’s instructions, and runs were accepted only when control values met the manufacturer’s acceptance criteria. To evaluate the concordance between sample types, plasma Aβ42 and Aβ40 levels were also measured in the paired heparinized plasma samples using the HISCL assay.

### 2.5. Statistical Analysis

Statistical analyses were performed using R software version 4.5.2 (R Foundation for Statistical Computing, Vienna, Austria). Continuous variables are presented as medians with interquartile ranges (IQRs). To evaluate the analytical concordance between assays, method comparison was conducted in accordance with the Clinical and Laboratory Standards Institute (CLSI) EP09c-Ed3 guidelines [17]; difference plots were generated to visualize agreement, and Passing–Bablok regression analysis was performed to assess the linear relationship and systematic bias, assuming a non-parametric distribution. For comparisons involving three or more groups, the Kruskal–Wallis test was employed, followed by post hoc analysis with Bonferroni correction for multiple comparisons. Diagnostic performance was evaluated using Receiver Operating Characteristic (ROC) curve analyses, with the Area Under the Curve (AUC) calculated for each biomarker. Pairwise comparisons of AUCs were performed using the DeLong test. For biomarker combination models, multivariable logistic regression was utilized to generate predicted probabilities.

## 3. Results

### 3.1. Participant Characteristics

A total of 127 participants were enrolled in this study. The median age of the participants was 78.0 years (IQR 72.0–83.0), and 56.7% were female. The group comprised 30.7% CN, 36.2% MCI, 14.2% AD, and 18.9% Non-AD participants (Table 1).

Regarding genetic risk factors, 39.3% of the participants were heterozygous or homozygous for the *APOE* ε4 allele, while 57.5% were non-carriers. Cognitive function was primarily assessed using the MMSE, with 48.0% of participants scoring within the normal range (≥24) and 22.0% showing severe impairment (≤18). Amyloid PET/CT imaging was available for only 11 participants.

### 3.2. Method Comparison Between HISCL and Simoa Assays

**Figure 1:**
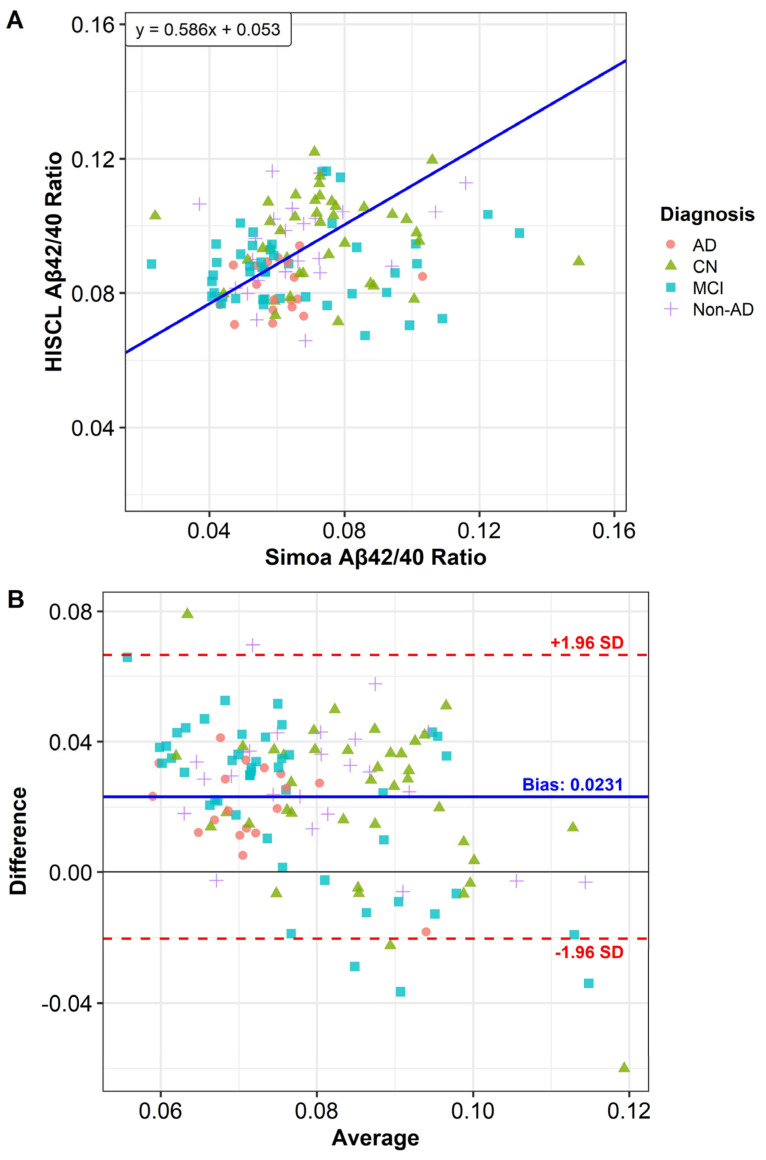
Method comparison between the HISCL and the Simoa Aβ42/40 ratio: (**A**) scatter plot with Passing–Bablok regression; (**B**) Bland–Altman plot. The solid blue line represents the mean difference (bias), and the dashed red lines indicate the 95% limits of agreement (mean ± 1.96 SD). Abbreviations: Aβ, amyloid-β; SD, standard deviation; CN, cognitively normal; MCI, mild cognitive impairment; AD, Alzheimer’s disease.

The comparative performance of the HISCL and Simoa assays was evaluated using difference plots (Figure 1A) and Passing-Bablok regression analysis (Figure 1B). The regression analysis indicated a proportional difference between the two platforms, yielding a slope of 0.556 (95% confidence interval (CI): 0.367 to 0.782) and an intercept of 0.055 (95% CI: 0.042 to 0.066). To investigate the source of the analytical discrepancy observed in the ratio, we performed a method comparison for the individual component assays. Passing–Bablok regression for plasma Aβ42 yielded a slope of 3.816 and an intercept of −3.296, with a mean bias of 9.73 pg/mL. For plasma Aβ40, the slope was 1.714, and the intercept was 25.455, with a mean bias of 81.04 pg/mL. These results indicated that the analytical discrepancy in the ratio was reflected in both individual components. We further analyzed the discordant cases (*n* = 26) based on the optimal cut-offs derived from ROC analysis. Only three participants were HISCL-positive/Simoa-negative, showing mixed diagnoses (one each of CN, MCI, and Non-AD). Conversely, 23 participants were HISCL-negative/Simoa-positive. Notably, in this group, the majority were clinically classified as CN (*n* = 14, 60.9%) or Non-AD (*n* = 6, 26.1%), with only three MCI cases (Table 2).

### 3.3. Diagnostic Performance of HISCL Plasma Aβ42/40 Ratio According to Clinical Diagnosis

**Figure 2:**
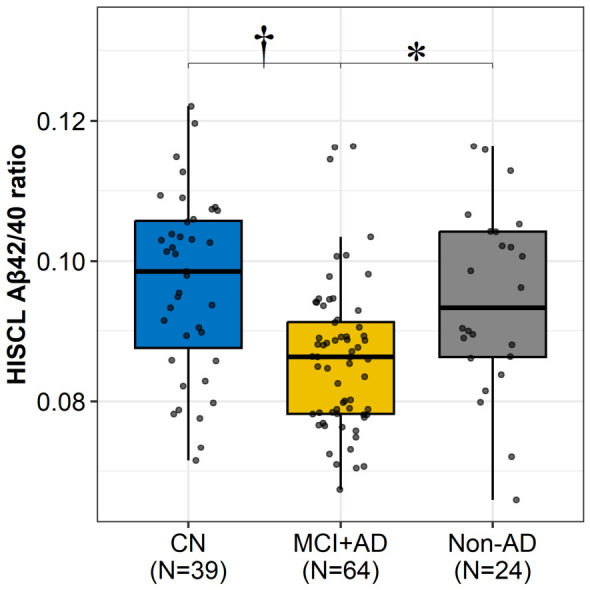
Kruskal–Wallis test according to clinical diagnosis. * *p* < 0.05, † *p* < 0.001. Abbreviations: CN, cognitively normal; MCI, mild cognitive impairment; AD, Alzheimer’s disease.

To evaluate the clinical validity of the HISCL assay, we analyzed plasma Aβ42/40 ratios across diagnostic categories. Considering the pathophysiological continuity and clinical relevance, patients with MCI and AD were combined into a single subgroup (MCI + AD) representing the Alzheimer’s disease continuum. The Kruskal–Wallis test indicated significant differences among the subgroups (Figure 2). Post hoc analysis revealed that the Aβ42/40 ratio was significantly lower in the MCI + AD subgroup (median 0.0863, 0.0782–0.0912) compared to the CN subgroup (median 0.0985, 0.0876–0.106; *p* < 0.001). Furthermore, the MCI + AD subgroup exhibited significantly lower ratios compared to the Non-AD subgroup (median 0.0933, 0.0863–0.104; *p* = 0.0099), whereas no significant difference was observed between the CN and Non-AD subgroups.

### 3.4. Comparison of Diagnostic Performance Among Plasma Biomarkers

**Figure 3:**
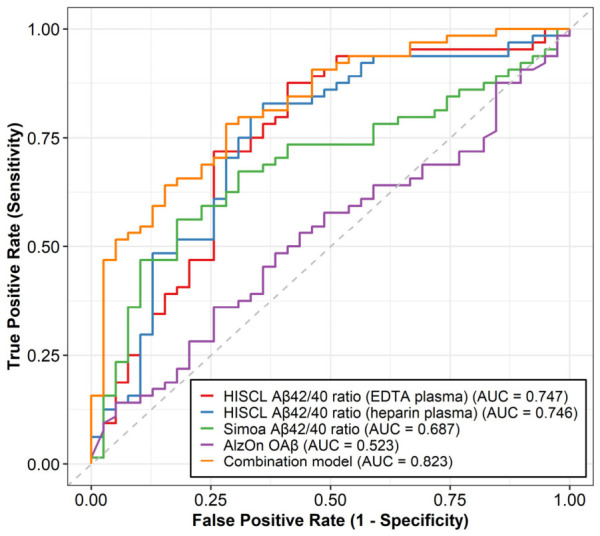
Receiver operating characteristic curves for discriminating the Alzheimer’s disease continuum from the cognitively normal subgroup. Abbreviations: Aβ, amyloid-β; OAβ, oligomerized amyloid-β; AUC, area under the curve.

To evaluate the diagnostic accuracy of various plasma biomarkers in identifying the AD continuum, ROC curve analyses were performed (Figure 3). The HISCL Aβ42/40 ratio measured in standard EDTA plasma, as recommended by the manufacturer, demonstrated robust diagnostic performance, with an AUC of 0.747. This was comparably higher than that of the Simoa Aβ42/40 ratio (AUC = 0.687, *p* = 0.392) and the OAβ assay (AUC = 0.523, *p* = 0.002). Notably, the HISCL assay performed in heparinized plasma (AUC = 0.746) showed a negligible difference from the EDTA results. Finally, the diagnostic performance was further enhanced when the HISCL Aβ42/40 ratio was integrated into a combination model with plasma GFAP and NfL levels, achieving the highest AUC of 0.823 (*p* = 0.046; in comparison to the HISCL Aβ42/40 ratio alone).

## 4. Discussion

In this study, we evaluated the diagnostic performance and clinical utility of the fully automated HISCL plasma Aβ42/40 assay in identifying the AD continuum within a real-world clinical setting. Our findings demonstrate that the HISCL assay effectively differentiates patients with MCI and AD from both cognitively normal controls and individuals with non-AD dementia, exhibiting robust diagnostic accuracy.

Method comparison analysis revealed a proportional difference between the HISCL and Simoa assays, suggesting differences in calibration between the two platforms. Nevertheless, this analytical discrepancy did not hinder the diagnostic potential of the HISCL assay; rather, it demonstrated a discriminative capability with a numerically higher AUC compared to the Simoa platform. While this difference in AUC did not reach statistical significance (*p* = 0.392), the HISCL platform offers distinct operational advantages. As a fully automated system, it provides high throughput, a rapid turnaround time, and low operational costs, making it suitable for routine clinical laboratory settings. Interestingly, the analysis of discordant results revealed distinct characteristics between the two platforms. The majority of discordant cases (*n* = 23) were positive by Simoa but negative by HISCL. Crucially, 87% of these participants (20 out of 23) belonged to either the CN or Non-AD groups, implying that the Simoa assay might be more susceptible to false-positive classifications in populations without Alzheimer’s pathology. In contrast, the HISCL assay demonstrated a higher concordance with the Non-AD status in these discrepant cases. In the context of a first-line screening tool, this high specificity is paramount; it minimizes unnecessary patient anxiety and prevents the medical costs associated with unwarranted follow-up PET scans or invasive CSF analyses for individuals who do not actually harbor cerebral amyloid pathology.

Notably, the HISCL assay maintained consistent diagnostic performance across different anticoagulant types, including heparinized plasma, as indicated in a previous report [18] and further supported by our data demonstrating a significant positive correlation and a regression slope near unity with negligible bias (Supplementary Figure S1). Considering that high accessibility and scalability are primary advantages of blood-based biomarkers, this flexibility in sample types represents a significant strength.

Recent literature emphasizes that AD is a multifactorial pathology; thus, a multi-biomarker approach—combining indicators of amyloidosis, tauopathy, and neurodegeneration—is essential for maximizing diagnostic precision and capturing the full complexity of the disease [19]. Consistent with this perspective, our study confirmed that integrating the HISCL plasma Aβ42/40 ratio with neurodegeneration-related biomarkers, specifically GFAP and NfL, significantly enhances predictive accuracy. To further investigate whether this synergistic effect is platform-specific, we additionally evaluated combination models for the Simoa and AlzOn assays. Similar improvements were observed, with the Simoa-based model reaching an AUC of 0.790 and the AlzOn-based model reaching an AUC of 0.767. These findings suggest that the multi-biomarker approach consistently improves diagnostic precision across different analytical platforms, with the HISCL-based combination achieving the highest numerical accuracy (AUC = 0.823). This synergistic improvement has important implications for the assay’s long-term clinical utility; it suggests that the HISCL Aβ42/40 assay can serve not only as a robust standalone screening tool but also as a foundational component of future combinatorial diagnostic panels. Consequently, even as novel biomarkers continue to emerge, this assay is likely to retain its value by contributing to more comprehensive and accurate risk stratification models. This is exemplified by the FDA-approved Lumipulse G pTau217/β-Amyloid 1-42 Plasma Ratio (Fujirebio Europe, Ghent, Belgium), which proved high performance in detecting cerebral AD pathologies [20].

Several limitations should be acknowledged. First, the retrospective design and the relatively small sample size—specifically, the limited number of participants in the AD subgroup (*n* = 18)—constitute a primary limitation. This constraint may reduce overall statistical power and limit the ability to fully capture the pathophysiological heterogeneity of the broader AD population, potentially affecting the generalizability of our optimal cut-off values. However, it is worth noting that our cohort size is comparable to that of numerous independent clinical validation studies aimed at evaluating plasma biomarker platforms [12,21,22,23,24]. While larger multi-center cohorts are ideal for establishing definitive reference intervals, our sample size proved sufficient to demonstrate statistically significant discriminative performance and to conduct method comparisons.

Second, the reliance primarily on clinical diagnosis rather than definitive amyloid PET or CSF biomarkers for patient classification is a notable weakness. While clinical diagnoses were made by experts, this approach carries an inherent risk of misclassification (e.g., non-AD dementia masking as AD, or underlying mixed pathologies). The limited availability of amyloid PET/CT imaging (*n* = 11) restricts the comprehensive validation of our assay against the neuroimaging gold standard. However, it is noteworthy that all six participants with confirmed amyloid PET positivity exhibited concordantly low plasma Aβ42/40 ratios. This observation aligns with recent findings in a Korean cohort [24], supporting the assay’s capability to reflect cerebral amyloid pathology despite the limited sample size.

Third, given the extended preclinical phase of the AD continuum, the lack of longitudinal follow-up limited our ability to monitor the temporal trajectory of biomarker changes. This limitation is particularly relevant regarding subjective cognitive impairment (SCI). With the growing recognition of SCI as a critical early indicator of neurodegeneration [25], a more granular classification that distinguished SCI from cognitively normal controls would have been valuable. Our inability to identify and separately analyze this high-risk subgroup—who are statistically more likely to progress to MCI according to recent studies [26,27]—may have obscured subtle biomarker elevations in the earliest stages of the disease, which a longer observation period or specific SCI screening could have captured.

Fourth, the Non-AD subgroup comprised a heterogeneous mix of non-Alzheimer pathologies, which could not be analyzed individually due to sample size constraints. For instance, previous research has demonstrated that reduced CSF Aβ42 levels in Parkinson’s disease with mild cognitive impairment (PD-MCI) are predictive of rapid progression to dementia [28]. In light of this, investigating the correlation between plasma Aβ42 levels and cognitive function, specifically within the PD cases, would have been of significant clinical interest. However, the limited number of PD cases in our study precluded such granular stratification.

Future research should focus on establishing harmonized cutoff values through large-scale, multi-center studies involving local populations. Furthermore, longitudinal investigations are warranted to determine whether baseline plasma Aβ42/40 levels can predict cognitive decline in preclinical stages.

Regarding its intended clinical role, we propose the HISCL assay primarily as a first-line screening tool in primary and secondary care settings, rather than a definitive replacement for amyloid PET or CSF analysis. By effectively identifying individuals with a high probability of AD pathology, this assay can triage patients, thereby reducing the number of unnecessary, invasive, and expensive confirmatory tests in cognitively normal individuals or those with non-AD etiologies. From a health economics perspective, it would be meaningful for future studies to investigate the impact of implementing the HISCL assay as a first-line screening tool; specifically, quantifying the resulting reduction in medical costs and the increase in patient compliance would provide critical evidence to support its role as an effective gatekeeper in stepwise diagnostic algorithms.

In conclusion, despite the acknowledged limitations, our study provides compelling real-world evidence supporting the clinical utility of the fully automated HISCL plasma Aβ42/40 assay. Its robust diagnostic performance, combined with analytical stability across sample types and high throughput capability, underscores its potential as a reliable screening modality. As the therapeutic landscape for AD evolves towards early intervention, we anticipate that this accessible blood-based biomarker will play a pivotal role in shifting the diagnostic paradigm to routine clinical practice.

## Figures and Tables

**Table 1:** Characteristics of study participants (Total *n* = 127).

Characteristics	*n* (%)
**Age (median 78.0, IQR 72.0–83.0)**	
<60	7 (5.5%)
60–69	20 (15.7%)
70–79	44 (34.6%)
80–89	49 (38.6%)
>90	7 (5.5%)
**Sex**	
Female	72 (56.7%)
Male	55 (43.3%)
**Clinical Diagnosis**	
CN	39 (30.7%)
MCI	46 (36.2%)
AD	18 (14.2%)
Non-AD	24 (18.9%)
***APOE*** **Genotype**	
E4 non-carrier	73 (57.5%)
E4 carrier (heterozygote)	46 (36.2%)
E4 carrier (homozygote)	4 (3.1%)
Unknown	4 (3.1%)
**MMSE Score**	
24–30	61 (48.0%)
19–23	25 (19.7%)
≤18	28 (22.0%)
Unknown	13 (10.2%)

Abbreviations: CN, cognitive normal; MCI, mild cognitive impairment; AD, Alzheimer’s disease; Non-AD, non-Alzheimer’s disease; MMSE, Mini-Mental State Examination.

**Table 2:** Diagnostic classification of discordant cases between HISCL and Simoa assays.

		**Simoa**			**Total**
**HISCL**		**Positive**	**Risk group**	**Negative**	
	**Positive**	66 (52.0%)	6 (4.7%)	3 (2.4%)	75 (59.1%)
	**Risk group**	19 (15.0%)	3 (2.4%)	2 (1.6%)	24 (18.9%)
	**Negative**	23 (18.1%)	1 (0.8%)	4 (3.1%)	28 (22%)
**Total**		108 (85%)	10 (7.9%)	9 (7.1%)	127 (100%)

## Data Availability

The data presented in this study are available on request from the corresponding author. The data are not publicly available due to privacy restrictions.

## Supplementary Materials

The following supporting information can be downloaded at: https://www.mdpi.com/article/10.3390/diagnostics16121767/s1, Figure S1: Comparison of EDTA and heparinized plasma for the HISCL Aβ42/40 assay. (A). Scatter plot with Passing-Bablok regression. (B). Bland-Altman plot. The solid blue line represents the mean difference (bias), the dashed red lines indicate the 95% limits of agreement (mean ± 1.96 SD). Table S1: Diagnostic classification of discordant cases between HISCL and Simoa assays.
