# Supplementary material for: Diagnostic Performance and Clinical Utility of Automated Plasma Amyloid-β 1-42/1-40 Assay
Source: Diagnostics (Basel). 2026 Jun 8;16(12):1767. doi: 10.3390/diagnostics16121767 (PMC13297981; doi:10.3390/diagnostics16121767)
Supplement: Supplementary file 1 [file diagnostics-16-01767-s001.zip › diagnostics-4321060-supplementary.pdf]

Table S1. Diagnostic classification of discordant cases between HISCL and Simoa assays.

	Diagnosis				Total
	CN	MCI	AD	Non-AD	
HISCL (+) / Simoa (-)	1 (33.3%)	1 (33.3%)	1 (33.3%)	0 (0%)	3
HISCL (-) / Simoa (+)	14 (60.9%)	3 (13%)	0 (0%)	6 (26.1%)	23

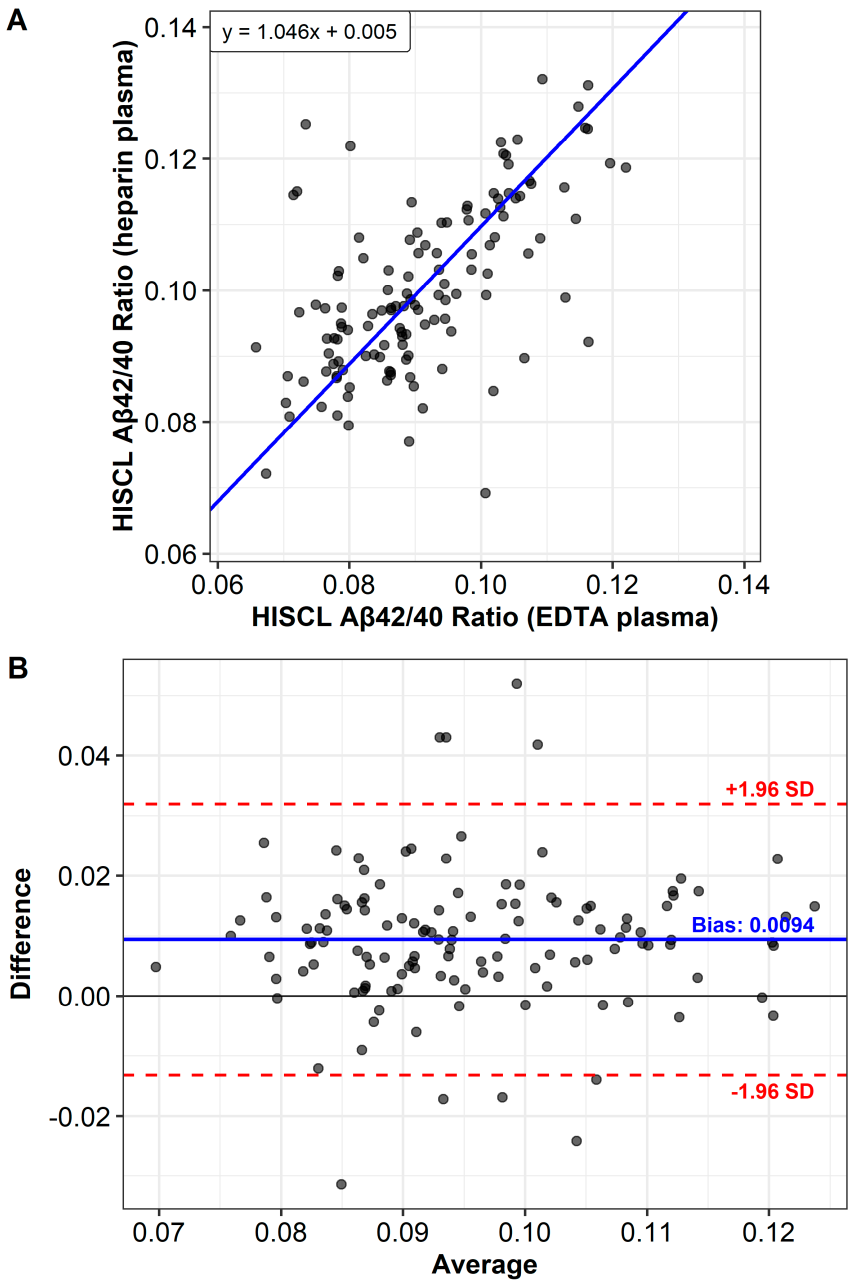

Figure S1. (A), scatter plot with Passing-Bablok regression. (B), Bland-Altman plot. The solid blue line represents the mean difference (bias), the dashed red lines indicate the 95% limits of agreement (mean ± 1.96 SD). Abbreviations: Aβ, amyloid-β; SD, standard deviation.
